# Conductive Biomass Films Containing Graphene Oxide and Cationic Cellulose Nanofibers for Electric-Heating Applications

**DOI:** 10.3390/nano11051187

**Published:** 2021-04-30

**Authors:** Shanqing Liang, Huicong Wang, Xin Tao

**Affiliations:** Research Institute of Wood Industry, Chinese Academy of Forestry, Beijing 100091, China; whchong95@163.com (H.W.); taushin1994@163.com (X.T.)

**Keywords:** graphene, cationic cellulose nanofiber, conductivity, electric-heating performance, power density

## Abstract

A low-voltage biomass matrix and flexible electric-heating composite with graphene oxide (GO) and cationic cellulose nanofiber (CCNF) were fabricated by ultrasonic dispersion and suction filtration. The main results show that the tensile strength and strain of the films decreased with an increase in the GO content, but the thermal stability increased. The GO/CCNF film underwent rapid thermal decomposition at 250–350 °C, and the maximum degradation temperature was higher by 19 °C compared to that of the pure CCNF film. It was found that the electrical conductivity increased from 0.013 to 2.96 S/cm with an increase in the GO content from 20 to 60 wt%, resulting in an increase in the power density from 122 to 2456 W/m^2^. The films could rapidly attain the temperature within 50 s, and the heat transferred by radiation and convection was 21.62 mW/°C, thereby exhibiting excellent electric heating response. Moreover, the film demonstrated a stable electric-heating cycle after a 12.5 h cycling test and meets the requirements of low-temperature electric heating products under the 36 V electric safety limit, which expands the potential applications of biomass-derived cellulose nanofibers.

## 1. Introduction

Electric-heating composites can convert electrical energy into heat energy in a controlled manner and have been widely used in many fields [1,2,3]. However, traditional electric-heating composites cannot meet the needs of the rapidly developed electrothermal products, owing to their disadvantages of low heat-transfer efficiency, complex preparation techniques, and non-flexibility. Currently, carbon materials are the preferred conductive materials for electric-heating composites because of their light weight, low voltage, oxidation resistance, rapid electric-heating response, and high heat-transfer efficiency. Carbon materials such as graphene, carbon nanotubes, and carbon fibers [4] have been used to synthesize electric-heating composites with applications in smart wear [5,6,7], heating and healthy clothing [8], deicing products [9], and electric-heating coatings [10]. Therefore, the study of carbon-based electric heating composites has become an interesting research direction. Some investigators have carried out extensive and in-depth studies on preparation methods, the selection of matrices, and functionalization.

Graphene is used to prepare low-voltage and high-efficiency electric-heating composites owing to its excellent electrical conductivity, thermal conductivity, high specific surface area, and mechanical properties. The preparation methods mainly include suction filtration, wet spinning, chemical vapor deposition (CVD), and spin coating [11,12]. For example, using polyethylene terephthalate (PET) as the matrix, graphene electrothermal films have been prepared by the CVD method, in which graphene was deposited on the surface of a PET substrate. The square resistance of the films was 159 Ω, and the maximum temperature was in the range 33–139 °C at a load of 5–30 V, and they exhibited a fast electric-heating response [13]. Similarly, good results were obtained for graphene-based electric heating films prepared by the wet spinning method. The steady-state temperatures of the films were in the range 33–177 °C after 2 min of loading voltage [14]. In addition, spin-coating and spraying methods have been used to prepare electric-heating films with quartz or flexible polyimide as the substrate. Furthermore, a fabric electric-heating material has been fabricated by the spraying method, and its steady-state temperature reached 162.6 °C at a load of 10 V, which proves that the graphene-based electric-heating composite has a high electric-heating efficiency [15].

In addition to the use of resin, quartz, and fabric as substrates, environmentally friendly cellulose (with advantages such as renewability, biocompatibility, non-toxicity, hydrogen-bonding capacity, sustainability) has attracted the attention of researchers, and the electrical, electrochemical, and electromagnetic characteristics of biomass-based composite films have been investigated [16,17,18,19]. Recently, research groups have reported the use of graphene and carbon nanotubes to prepare degradable electric heating composites by their impregnation on cellulose paper or mixing with cellulose. The results demonstrated several advantages including high electrical conductivity, rapid electrothermal effect, and low energy consumption [20,21]. However, nanocellulose-based films or gels prepared using cellulose microfibrils, microcrystalline cellulose, cellulose nanocrystals, cellulose nanofibrils, and bacterial cellulose show different electrical conductivities, which directly affect the electric properties of the composites [22,23], and cationic nanocellulose and anionic GO have been investigated and shown to be beneficial to the dispersion and reinforcement of composite materials [24,25,26]. It is very important to study the properties of electrothermal composites prepared from various types of nanocellulose, to obtain more reliable results and further promote the practical applications of nanocellulose-based electric-heating composites. 

In this paper, we present an eco-friendly electric-heating composite of GO and CCNF synthesized via a simple ultrasonic dispersion and vacuum filtration method. The effects of different GO contents on the mechanical properties, thermal stabilities, and electric-heating performances of the GO/CCNF films were characterized via micromechanical testing, scanning electron microscopy (SEM), electrothermal performance testing, and Raman spectroscopy. The electric-heating response and electric-to-radiant power transfer efficiency were analyzed thoroughly to provide a reference for the application of biomass-derived nanocellulose-based electric-heating composites.

## 2. Materials and Methods

### 2.1. Materials

An aqueous dispersion of graphene oxide (TNWPRGO), with a GO content of 1.30 wt% (dispersant content 0.3 wt%), purity > 98 wt%, thickness of 0.5–3.7 nm, median diameter D(50) of 4–6 μm, number of layers < 10, and surface area of 500–700 m^2^/g, was purchased from Chengdu Organic Chemistry Co., Ltd. (Chengdu, China), Chinese Academy of Sciences. Aqueous cationic cellulose nanofibers were procured from Tianjin Woodelf Biotechnology Co., Ltd. (Tianjin, China), with a CCNF content of 1.17 wt%, diameter of 10–15 nm, length of 1–5 μm. Anhydrous ethanol (purity 99.7%) was purchased from Sigma–Aldrich (St. Luis, MO, USA). Conductive silver glue, copper wire (diameter 0.12 mm), polytetrafluoroethylene (PTFE) membrane (hydrophilic type, diameter 110 mm, and pore size 0.22 μm.) were obtained from a market. All materials and chemicals were used as received, without further purification.

### 2.2. Preparation of GO/CCNF Films

**Figure 1:**
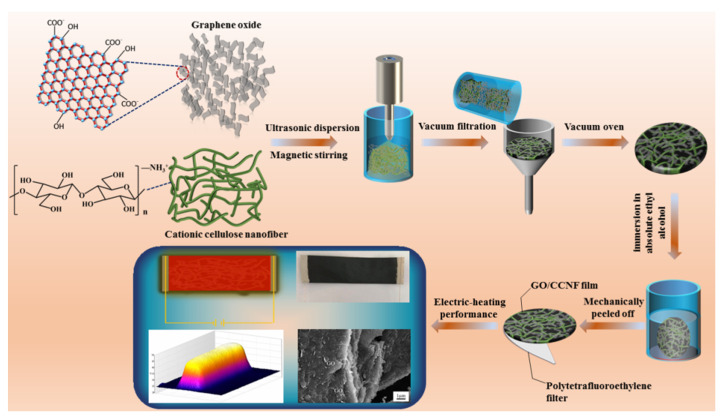
A schematic for preparing method and testing electric-heating performance of GO/CCNF composite films.

GO/CCNF films with different GO contents were prepared sequentially. Initially, GO (mass ratios: 1, 5, 10, 20, 30, 40, 50, and 60%) and CCNF aqueous dispersions were mixed in a 100 mL glass beaker. The solid content of the mixed GO and CCNF was 0.58 g. Then, 50 g distilled water was added and the mixture was subjected to sonication using an ultrasonic generator at 600 W for 5 min (water bath) followed by magnetic stirring for 30 min (1500 rpm). The mixture was quickly poured into a Buchner funnel with a polytetrafluoroethylene filter and filtered via vacuum filtration for 5 h. The films were placed with the polytetrafluoroethylene filter and dried in a vacuum oven at 60 °C for 2 h. Finally, the films were mechanically peeled off from the polytetrafluoroethylene filter by immersion in absolute ethyl alcohol for 2 min and were dried at room temperature (Figure 1).

### 2.3. Characterization

The GO/CCNF films were characterized via mechanical testing, SEM, and thermogravimetric analysis (TG and DTG). Samples of pure CCNF, GO/CCNF–20%, GO/CCNF–40%, and GO/CCNF–60% of 60 mm × 2 mm each were prepared and placed at room temperature for 1 week. Subsequently, their stress–strain properties were tested at a tensile speed of 0.5 mm/min (Shimadzu Autograph AGS–X, Shimadzu, Tokyo, Japan). The microstructures of the films were observed using a scanning electron microscope (Hitachi S4800, Hitachi, Tokyo, Japan) at a voltage of 10 kV. Further, the thermal stability of 20 mg samples of pure CCNF, GO/CCNF–1%, GO/CCNF–5%, GO/CCNF–10%, GO/CCNF–20%, GO/CCNF–40%, and GO/CCNF–60% was analyzed from 20 ℃ to 600 ℃ under nitrogen protection and a heating rate of 10 °C/min (STA 449F3, Netzsch Synchronous Thermal Analyzer, Gebrüder, Wuppertal, Germany).

### 2.4. Electric-Heating Performance 

The GO/CCNF films were cut to dimensions of 60 mm × 15 mm, and a copper wire, as the electrode, was bonded to each sample using a conductive silver glue. The electrode separation distance was 50 mm, and the films were dried in a vacuum oven at 60 °C for 1 h. The resistance, current–voltage (I–V), and electric power–voltage (P–V) correlations of the GO/CCNF films were investigated using a digital multimeter (F15B+, Fluke Co., Ltd, Everett, WA, USA), a voltage regulator (TDGC2–1000 V, Delixi, Shanghai, China), and an electrical parameter tester (WT310HC, Yokogawa, Tokyo, Japan). The electrical conductivity and electric power of the films were calculated according to the equation *σ* = *L/RS* (σ: electrical conductivity, S/cm; *L*: electrode separation distance, mm; *R*: resistance, Ω; *S*: cross-sectional area of the sample, mm^2^) and *P* = *IV* (*P*: electric power, *W*; I: current, A; *V*: voltage, V).

The electric-heating performance was investigated using a multi-channel temperature recorder (34972A, Agilent, Rocklin, CA, USA) and an electrical parameter tester (WT310HC, Yokogawa, Tokyo, Japan) to understand the temperature growth and cooling of films at different voltages. The power-on heating time was 500 s, and the power-off cooling time was 200 s. Infrared images were obtained using an infrared thermal imager (Ti100, Fluke, Everett, WA, USA). 

The electric-heating stability of the GO/CCNF composites with 40% GO was investigated using a heating and cooling cycle of 45 V (power-on) to heat for 1000 s, followed by cooling (power-off) for 500 s. The cycling was performed for a duration of 12.5 h. After electrothermal cycling, the films were subjected to Raman spectroscopy (inVia, Renishaw, London, UK) at a wavelength of 633 nm to analyze the peak changes.

## 3. Results

### 3.1. Mechanical Properties and Microstructure 

**Figure 2:**
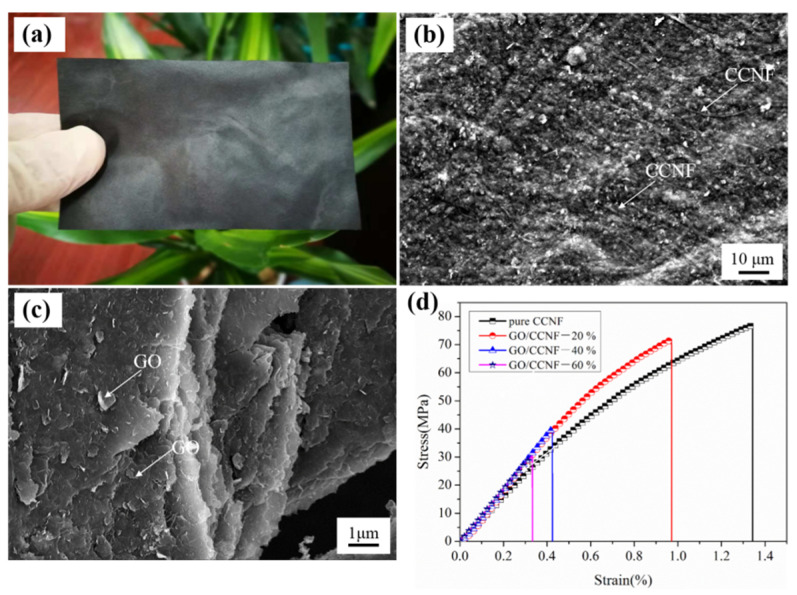
Mechanical properties and microstructure of GO/CCNF films. (**a**) sample photograph; (**b**) surface SEM image; (**c**) tensile section SEM image; (**d**) stress–strain curves.

Figure 2 shows photographs of the GO/CCNF films, SEM images, and partial tensile strength results. The SEM image shows that GO was uniformly distributed inside the electrothermal composite. With an increase in the amount of GO, the contact points between the GO also increase, thereby transforming the insulating film into a conductive composite. This shows that ultrasonic dispersion can effectively mix GO and CCNF, without significant agglomeration of GO, and the original structure was not damaged (Figure 2a–c). According to the stress–strain curves (Figure 2d), the fracture strength and strain of the pure CCNF film were 76.82 MPa and 1.34%, respectively. Conversely, with an increase in the GO content, i.e., in the films with 20, 40, and 60 wt% GO, the fracture strength and strain decreased to 71.83 MPa and 0.97%, 40.09 MPa and 0.42%, 30.47 MPa and 0.33%, respectively. Compared with that of the CCNF film, the tensile strength of the 20, 40, and 60 wt% GO films decreased by 6.50, 47.81, and 60.34%, respectively. Similarly, there was a more significant decrease in the strain of these samples, by 27.61, 68.66, and 75.37%, respectively. These results confirm that the brittleness of the GO/CCNF films increases significantly with an increase in GO. The tensile fracture clearly proves that GO is well-distributed in the composite. However, the mechanical strength of the films is mainly due to the combined effect of the forces between molecules and hydroxyl bonding. When the amount of GO was increased, the contact surface between the GO molecules increased, although the composite retained the mechanical properties of the cellulose nanofiber membrane [27,28,29]. However, when the GO changed from being wrapped by nanocellulose to being partially connected, and cellulose nanofibers did not form a chemical bond, the mechanical performance deteriorated, and the films exhibited brittle fractures during the stretching process.

### 3.2. Thermal Stability

**Figure 3:**
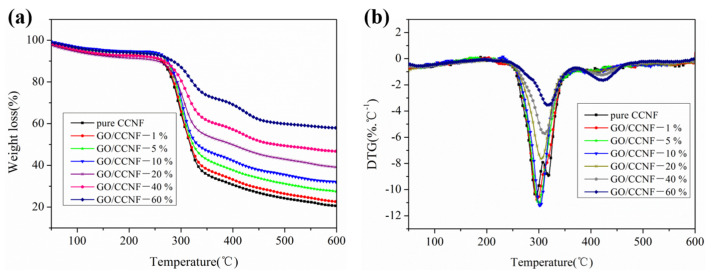
Thermal stability of GO/CCNF films. (**a**) TG curves; (**b**) DTG curves.

Figure 3 shows the TG and DTG curves of the GO/CCNF films. The thermal decomposition of the films can be mainly divided into initial decomposition, main decomposition, and residual decomposition. The first stage (25–250 °C) was that of slow degradation due to moisture volatilization and partial cellulose activation. In this stage, cellulose begins to get activated to form active groups, such as carbonyl and carboxyl groups, and produces CO_2_ and other gases, with a weight loss of approximately 15%. In the main decomposition stage (250–350 °C), the GO/CCNF films were rapidly pyrolyzed and increased the maximum weight loss temperature. For instance, the maximum degradation temperature was increased by 19 °C with 60 wt% GO compared with that of pure CCNF, which indicates that the heat stability of the films was improved with the addition of GO. In this stage, the weight loss was 64.33, 61.85, 57.18, 52.93, 45.71, 38.44, and 26.44%, respectively. The rapid degradation is due to the presence of low-molecular weight solvents and cellulose pyrolysis. Moreover, the DTG curves of the films show that the maximum weight loss temperature gradually increased with GO content, and after heating over 350 °C is the slow carbon residue process, and the residual mass mainly comprised undecomposed GO and cellulose residue. It is evident that an increase in the GO content improves the thermal stability of the films. However, because of the use of cellulose nanofibers as the matrix, the films should not be exposed to excessively high temperatures, as it may cause thermal degradation during application.

### 3.3. Electrical Conductivity

**Figure 4:**
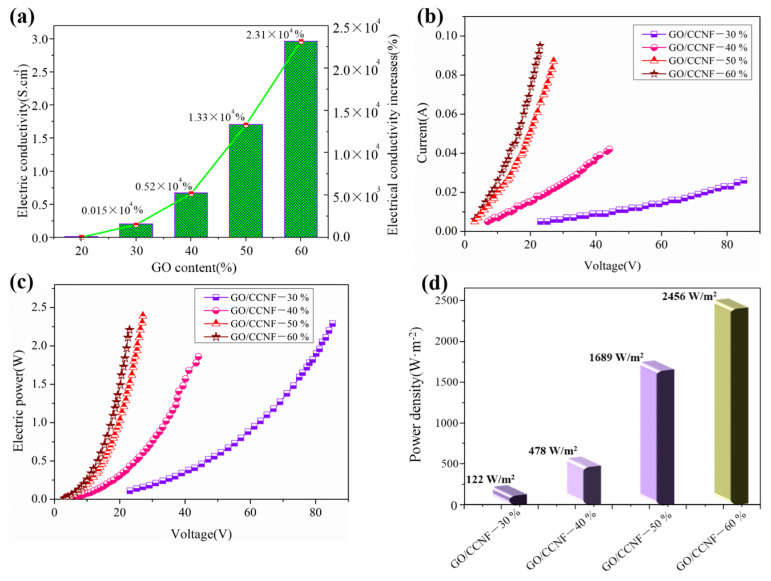
Electrical properties of GO/CCNF films. (**a**) conductivity; (**b**) voltage–current curves; (**c**) power–voltage curves; (**d**) power density.

Figure 4 shows the electrical conductivities of the GO/CCNF films. We found that GO addition significantly improved the electrical conductivity of the films. The films changed from being insulating composites to conductive composites because the GO promoted the formation of more conductive networks in the films. After the GO content was increased from 20 wt% to 60 wt%, the electrical conductivity of the films increased rapidly from 0.013 S/cm to 2.96 S/cm. Compared with the 20 wt% film, there was an increase in the electric conductivity of the 30, 40, 50, and 60 wt% films by 0.015 × 10^4^, 0.52 × 10^4^, 1.33 × 10^4^, and 2.31 × 10^4^%, respectively (Figure 4a). This shows that the addition of more than 20 wt% GO improved the electrical conductivity of the films. The purpose of preparing GO/CCNF films is to convert electrical energy into heat energy. Therefore, the correlation between current–voltage and electric power–voltage characteristics was analyzed mainly for the films with 30–60 wt% GO. The current–voltage of the films is clearly closely related. The values of current through the 30–60 wt% GO films were 0.005, 0.018, 0.066, and 0.095 A with a load of 23 V (Figure 4b), indicating that the current through the composites has a greater effect on the voltage. The higher the GO content, the higher is the current. These results are mainly because the current is directly proportional to the electric conductivity under the same applied voltage [30,31]. Furthermore, the electric power is proportional to the square of the voltage under the same resistance conditions. With a load of 23 V, the values of power of the films with 30–60 wt% GO were 0.11, 0.43, 1.52, and 2.21 W (Figure 4c). The power density increased from 122 W/m^2^ to 478, 1689, and 2456 W/m^2^ (Figure 4d). Generally, electrical power is inversely proportional to the resistance; hence, the higher the GO content, the better the power performance of the composite.

### 3.4. Electric-Heating Performance

**Figure 5:**
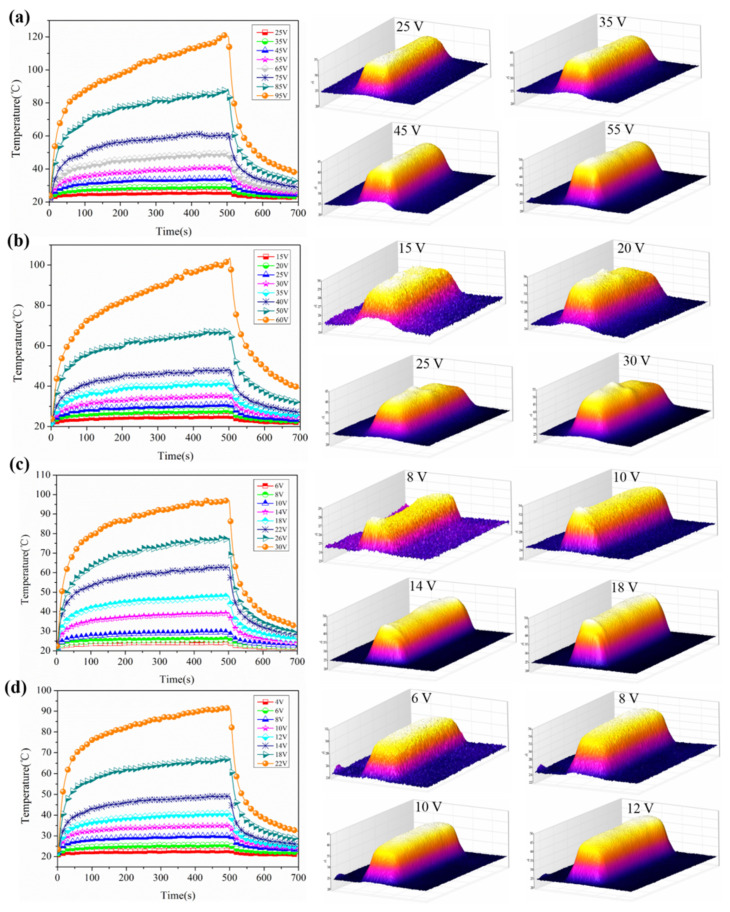
Time–temperature curves and infrared images of GO/CCNF films. (**a**) GO/CCNF–30%; (**b**) GO/CCNF–40%; (**c**) GO/CCNF–50%; (**d**) GO/CCNF–60%.

The time–temperature curves of the GO/CCNF films with 30–60 wt% GO are shown in Figure 5. The curves are divided into three stages: temperature growth, temperature stabilization, and temperature decline. After applying the load voltage, the temperature of the GO/CCNF films increased rapidly, within 50 s. In this time, the temperature of GO/CCNF–30% at 25–95 V reached 94.6, 90.3, 86.1, 82.0, 78.0, 74.9, and 69.2% of the maximum temperature (Figure 5a). The temperature stabilization stage can be attained rapidly at a lower voltage. Some of the films continued to generate Joule’s heat after a load of 50 s, and the temperature gradually reached equilibrium after a loading voltage of 100 s. The maximum temperatures of the film with 40 wt% GO are the range 25.2–103.6 °C at 15–60 V (Figure 5b). The electric heating response of the films is more sensitive after GO continued to increase to 50 and 60%; the heating temperature upon heating for 50 s can reach 72.1–95.1% and 75.2–95.5% of the maximum temperature loading of 6–30 V and 4–22 V, respectively (Figure 5c–d). For example, when the 30, 40, 50, and 60 wt% films reached the temperature of 91.5 °C, the loading voltages were 95, 60, 30, and 22 V, respectively. Hence, to achieve the same heating temperature, a lower voltage is required for samples with a higher GO content. After the voltage application was stopped, the films entered a temperature attenuation stage, and the temperature quickly dropped to the initial temperature. Furthermore, the infrared images of the GO/CCNF films show that the temperature distribution has no obvious high-temperature area and proves that the temperature distribution is relatively uniform, indicating that the GO is uniformly distributed in the cationic cellulose nanofiber matrix. Moreover, electrical safety is one of the key influencing factors in the practical application of electric heating products: an operating voltage below 36 V meets the requirements of electrical safety, especially in the field of wearable products and indoor heating furniture. The maximum temperatures should typically be in a relatively low temperature range for safety; thus, the GO amount and voltage range can be selected accordingly for different applications [32,33].

**Figure 6:**
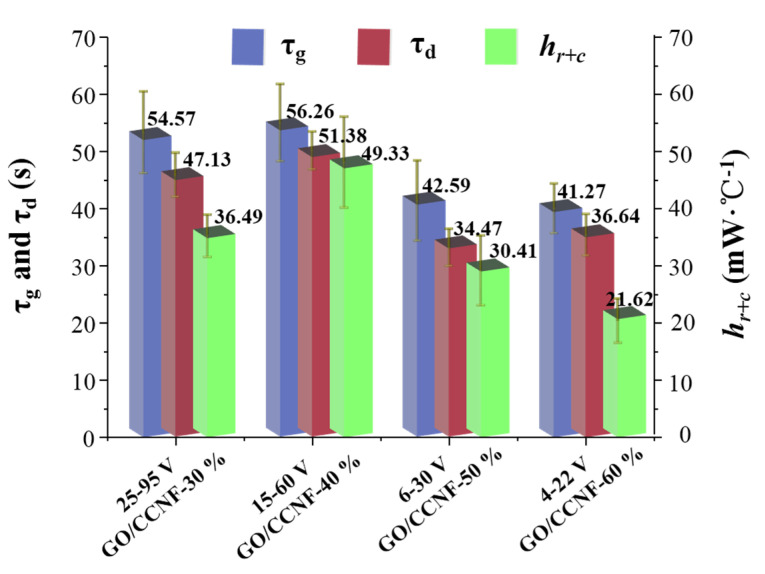
Electric-heating parameters of GO/CCNF films.

The characteristic growth time constant ($\;\tau_{g}$), decay time constant ($\tau_{d}$), and heat transferred by radiation and convection (*h*_r+c_) are used to explain the electric heating behavior of electrothermal composites based on the three stages in the time–temperature curves (Figure 5) [20,30,34]. In the three temperature regions,$\;\tau_{g},$ $\tau_{d}$, and *h*_r+c_ can be described by the following empirical formula:

(1) $$\left(\frac{T_{t}-T_{0}}{T_{m}-T_{0}}\right)=1-\exp\left(-\frac{t}{\tau_{g}}\right),$$

(2) $$\left(\frac{T_{t}-T_{0}}{T_{m}-T_{0}}\right)=\exp\left(-\frac{t}{\tau_{d}}\right),$$

(3) $$h_{r+c}=\frac{I_{c}V_{0}}{T_{m}-T_{0}}$$

where *T_0_* is the initial temperature, *Tm* is the maximum temperature, *Tt* is an arbitrary temperature at time *t*, *Ic* is the steady-state current, and *V_0_* is the initial applied voltage. Figure 6 shows the electric heating parameters of GO/CCNF films as derived from the equations. The film exhibited a fast temperature response performance for smaller values of$\;\tau_{g}\;$and$\;\tau_{d}$, indicating that the shorter the time taken for the electric heating composite to heat from the starting temperature to the maximum temperature, lesser is the time required to cool down from the maximum temperature. The $\tau_{g}\;$range was 41.27–56.26 s and the average value with 30–60 wt% GO addition was 48.67 s. This further proves that the fastest temperature increase time of the GO/CCNF film was approximately 50 s; the higher the amount of GO added, the more beneficial the rapid electric-heating response. The $\tau_{d}$ value of films varied with time in the range of 34.47–47.13 s, indicating that the maximum temperature drops to the initial temperature in a short time after stopping voltage, which is beneficial to the heating–cooling cycle response. Furthermore, the *h_r+c_* value is a key indicator that is characteristic of the conversion efficiency of an electric heating film in converting electrical energy to thermal energy. The lower the electric power consumed by the film with every increase of 1 °C, the higher is the electric-to-radiant power transfer efficiency. The average *h_r+c_* value of GO/CCNF films with 30 wt% GO is 36.49 mW/°C under a load voltage in the range of 25–95 V. When the GO increases to 60 wt%, its average *h_r+c_* value is 21.62 mW/°C using 4–22 V. The *h_r+c_* is considerably improved with an increase in GO and requires a lower voltage, which proves that the composite prepared in this study can be used to produce low-voltage and high-efficiency electric-heating products.

### 3.5. Electric-Heating Cycle Performance

**Figure 7:**
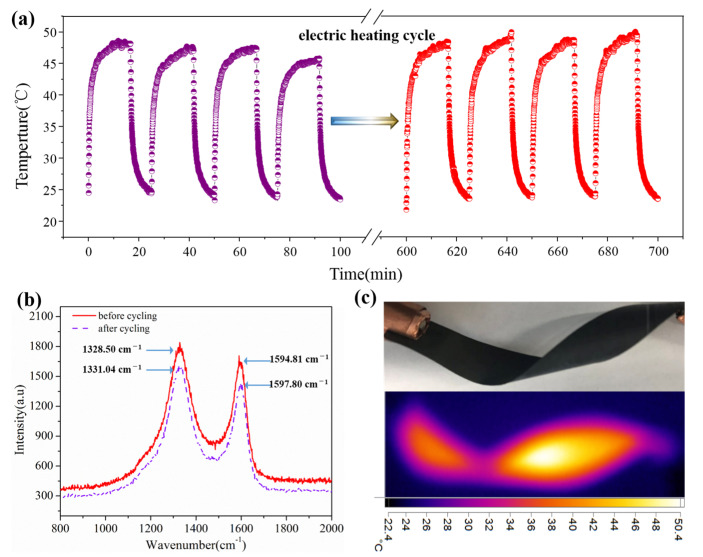
Electric-heating cycle performance of GO/CCNF film. (**a**) heating and cooling cycle; (**b**) Raman spectra; (**c**) flexibility.

To analyze the electric-heating cycle performance of the GO/CCNF films, the cycle performance of GO/CCNF–40% was tested for 12.5 h for heating and cooling cycles at a load voltage of 45 V. The electric-heating stability of the film did not exhibit an attenuation phenomenon. However, there is a certain difference in the maximum temperature of each cycle, with maximum temperatures in the range of 45.76–49.97 °C. Furthermore, the maximum temperature difference of the heating film is 4.21 °C during the 12.5 h heating and cooling cycle (Figure 7a). This is probably because of the environmental change in the heat radiation exchange and voltage deviation of the test system, but the electric-to-radiant power transfer efficiency of the composite was not affected. The results show that the film has a stable electric-heating cycle performance and exhibits good flexibility after the electric-heating cycle. In addition, Raman spectroscopy results show that the D-peak and G-peak before the electric-heating cycle were 1328.50 and 1594.81 cm^−1^. In contrast, the D-peak and G-peak after electric heating cycle were 1331.04 and 1597.80 cm^−1^ (Figure 7b). It is known that the D-peak represents the defects and disordered structure in the composite, and the intensity of the G-peak is determined by the degree of structural disorder. Furthermore, the ratio of the two characteristic peaks, I_D_/I_G_, represents the defect density of the composite. The I_D_/I_G_ of the GO/CCNF film was 0.83 before the electric-heating cycle and after cycling; that is, the degree of disorder of the carbon atoms in the film and the size of the existing defects did not change. Moreover, the oxygen-containing functional groups and the order of the sp^2^ type carbon structure were not affected after the electric heating cycle, enabling the electric heating film to maintain a stable electric-heating cycle performance and flexibility (Figure 7c) [31].

## 4. Conclusions

GO/CCNF films were successfully prepared, and their structural morphology, tensile strength, electrical conductivity, electric-heating performance, and cycle stability were investigated. GO was found to be uniformly distributed in the films, and the amount of GO affected their tensile properties and conductive network. The films were transformed from an insulating material to a 2.96 S/cm conductive composite. The power density of the films increased from 122 to 2456 W/m^2^ as the GO content increased from 20 to 60 wt%. The heating temperature of the GO/CCNF films increased rapidly, within 50 s, and the temperature gradually reached equilibrium after 100 s. The average temperature growth coefficient was 48.67 s, which indicates that the GO/CCNF film has sensitive electric heating response characteristics. The electric-to-radiant power transfer efficiencies of films with different contents of GO/CCNF were in the range of 21.62–36.49 mW/°C. In addition, after a 12.5 h electric–heating cycle, it was found that the film demonstrated a stable electric-heating performance and flexibility, which can be used to fabricate low-voltage and high-efficiency electric-heating products.

## Data Availability

The data presented in this study are available on request from the corresponding author.
